# S-100 Immunohistochemical Positivity in Rhabdomyoma: An Underestimated Potential Diagnostic Pitfall in Routine Practice

**DOI:** 10.3390/diagnostics12040892

**Published:** 2022-04-02

**Authors:** Andrea Palicelli, Antonio Ramponi, Guido Valente, Renzo Boldorini, Annalisa Balbo Mussetto, Magda Zanelli

**Affiliations:** 1 Pathology Unit, Azienda Unità Sanitaria Locale—IRCCS di Reggio Emilia, 42123 Reggio Emilia, Italy; magda.zanelli@ausl.re.it; 2 Pathology Unit, Department of Health Sciences, Università del Piemonte Orientale (UPO), 28100 Novara, Italy; antonio.ramponi.c6ac@no.omceo.it (A.R.); renzo.boldorini@med.uniupo.it (R.B.); 3 Pathology Unit, Department of Translational Medicine, “Sant’Andrea” Hospital, Università del Piemonte Orientale (UPO), 13100 Vercelli, Italy; guido.valente@med.uniupo.it; 4 Radiology Department, Umberto I Mauriziano Hospital, 10128 Turin, Italy; abalbomussetto@mauriziano.it

**Keywords:** rhabdomyoma, adult, larynx, head and neck, immunohistochemistry, S-100, S100, diagnosis, pitfalls, granular cell tumor

## Abstract

A 66-year-old man presented with a 2.8 cm lesion of the left vocal cord. On contrast-enhanced computed tomography scans, the tumor extended to the supraglottis, subglottis, paraglottic space and anterior commissure, causing partial obstruction of the laryngeal lumen. At another hospital, a fragmented incisional biopsy was diagnosed as a granular cell tumor, as to the S-100 immunohistochemical positivity. After excision, the tumor revealed to be an adult-type laryngeal rhabdomyoma. The typical cytoplasmic rod-like inclusions and cross striations were more evident in the second specimen. We confirmed the unusual S-100 immunohistochemical positivity (variable intensity, >90% of tumor cells). Muscle markers were not performed on the previous biopsy, resulting positive in our specimen (Desmin: strong, diffuse expression; Smooth Muscle Actin: strong staining in 10% of tumor cells). Melan-A, CD68, GFAP, pan-cytokeratins, CEA, calretinin and neurofilaments resulted negative. To our brief, systematic literature review, S-100 positivity (usually variable, often weak or patchy/focal) was globally found in 19/34 (56%) adult-type rhabdomyomas of the head and neck region. Especially on fragmented biopsy material, the differential diagnoses of laryngeal rhabdomyomas may include granular cell tumors, oncocytic tumors of the salivary glands or of different origin, and paragangliomas.

**Figure 1:**
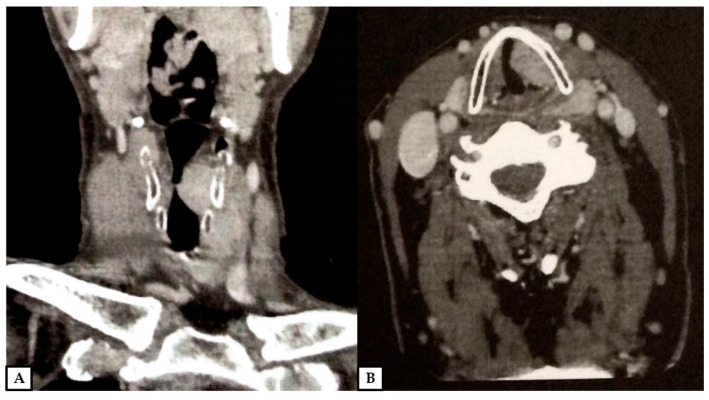
A 66-year-old man presented with a previous diagnosis of granular cell tumor of the left vocal cord on an incisional biopsy performed at another hospital. Contrast-enhanced computed tomography revealed a soft tissue mass of 2.8 × 2.5 × 1.1 cm of the left vocal cord, with transglottic extension, spreading to the supraglottis, subglottis, paraglottic space and the anterior commissure, causing partial obstruction of the laryngeal lumen; no infiltration of the cartilage was evident (computed tomography scans; (**A**): coronal plane, (**B**): axial plane; previously unpublished, original photos).

**Figure 2:**
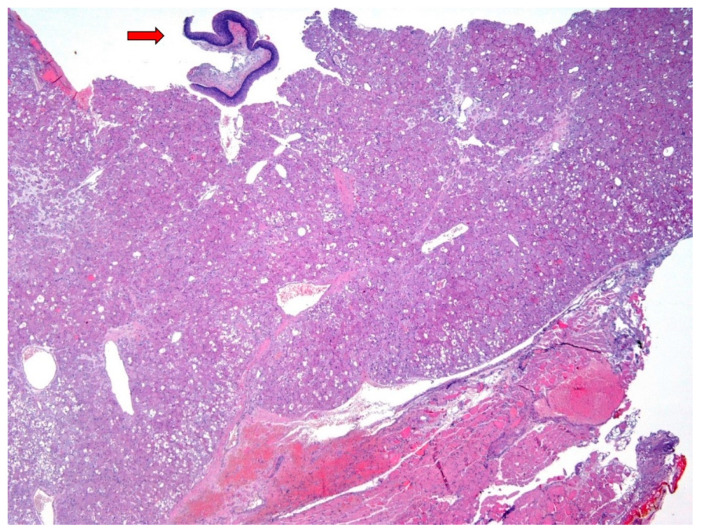
An excisional biopsy was performed. On gross exam, the lesion had a hemorrhagic appearance. Histological examination revealed a highly cellular tumor (upper part of the Figure), involving the subepithelial connective tissue, with extensive superficial de-epithelization (arrow: residual squamous epithelium of the vocal cord). The lesion was unencapsulated and mainly well-delimited from the underlying striated muscle layer (lower part of the Figure), with pushing growth borders; however, the partial fragmentation of the material did not allow a complete definition of the tumor growth front. The surgical margins were involved, while biopsies of the bilateral false vocal cords were unaffected (Hematoxylin and Eosin, 4×; previously unpublished, original photo).

**Figure 3:**
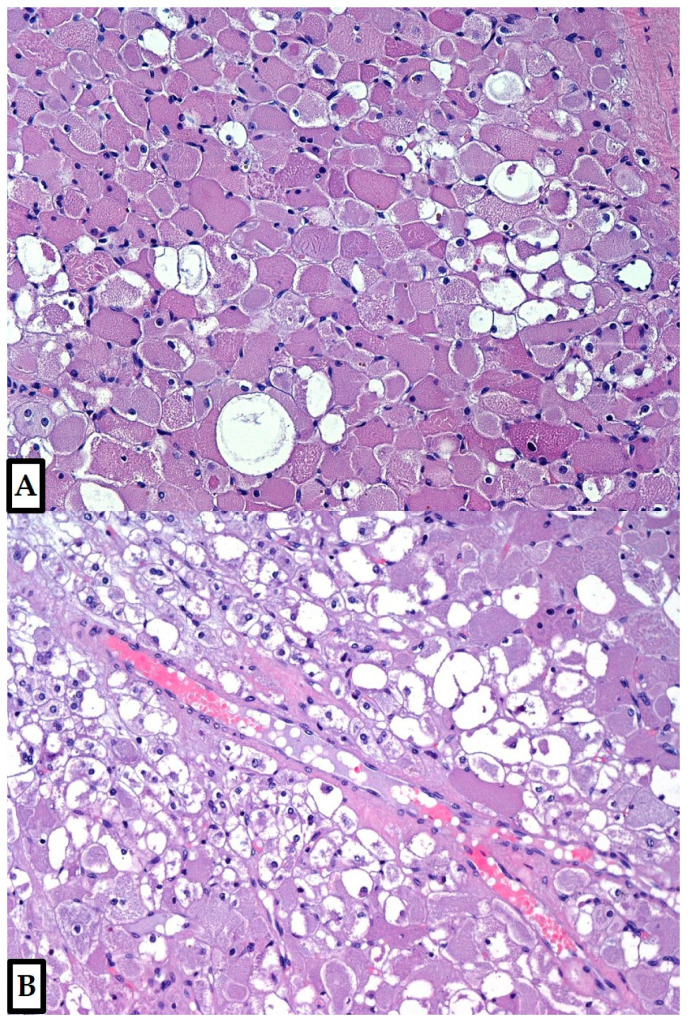
The tumor was composed of large polygonal cells of variable size and shape, with abundant granular eosinophilic cytoplasm; vacuolated cells (spider cells) were usually dispersed through the tumor (**A**), occasionally concentrated in some areas (**B**). Vascularization included small, sometimes dilated capillaries and occasional ectatic medium-sized vessels (Hematoxylin and Eosin, 10×; previously unpublished, original photos).

**Figure 4:**
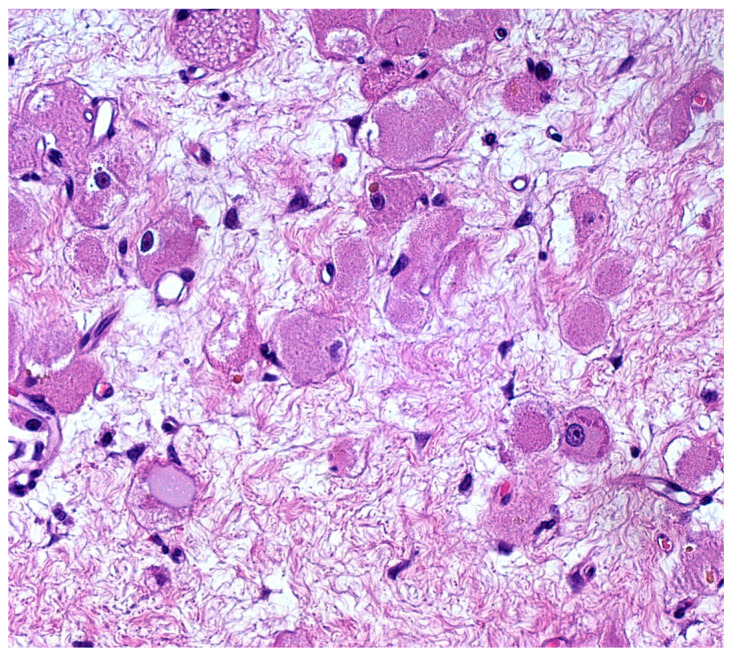
Nuclear atypia was absent to mild. Indeed, the large- to medium-sized tumor cells showed relatively monomorphic, peripherally or centrally located, vesicular nuclei of small to medium size; the chromatin was fine, despite occasional slight variation in nuclear size and shape, with irregularities of nuclear membranes and rare multinucleations. The nucleoli were occasionally evident, but small. Pleomorphism, mitoses and necrosis were absent. The intercellular stroma was almost virtual in the majority of the tumor, but loose connective tissue (no desmoplasia) was found in less-cellular areas (like this one) (Hematoxylin and Eosin, 20×; previously unpublished, original photo).

**Figure 5:**
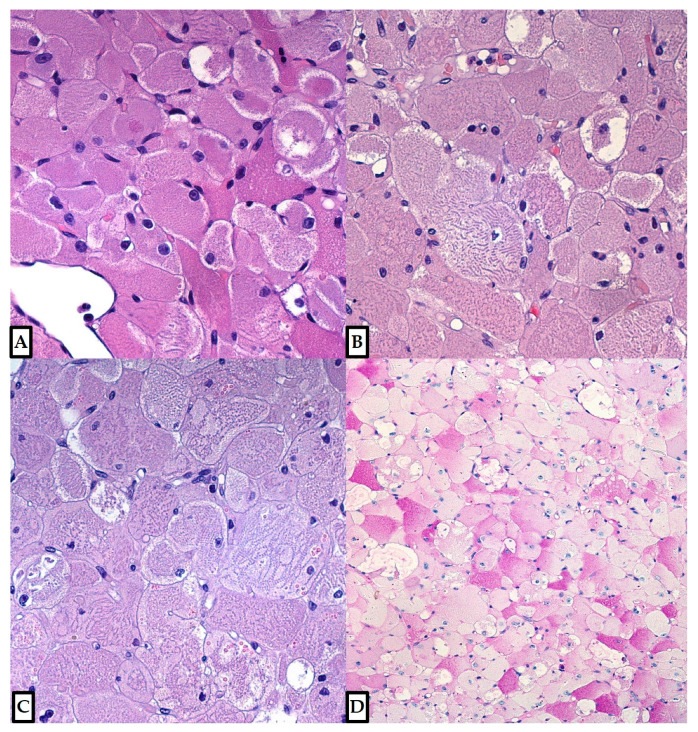
On higher power, cytoplasmic rod-like inclusions and often subtle cross striations were present in many cells ((**A**–**C**); Hematoxylin and Eosin, 20×; previously unpublished, original photos), being more subtle in some areas (as in Figure 5A). Periodic Acid–Schiff (PAS) stain showed variable cytoplasmic positivity ((**D**); 10×; previously unpublished, original photo), while PAS-diastase reaction resulted negative.

**Figure 6:**
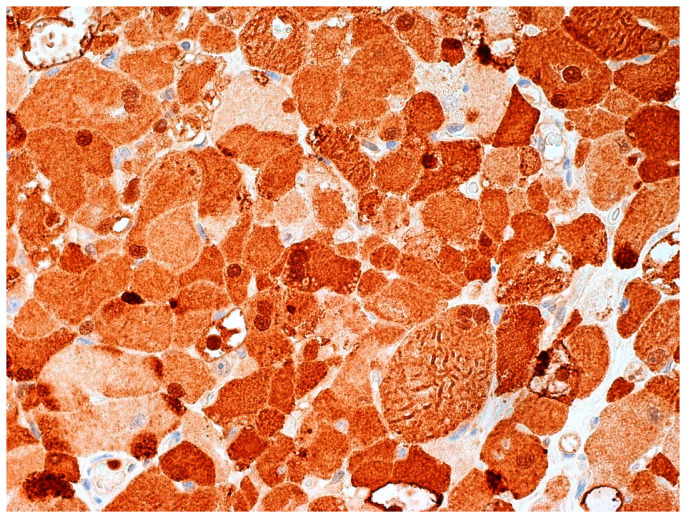
As the case had the morphological features of an adult-type rhabdomyoma (RM), previous slides were retrieved. Rod-like inclusions and cross striations were less evident and the material was fragmented; S-100 immunohistochemical expression was previously reported. Indeed, S-100 positivity was confirmed in >90% of tumor cells in our specimen, which showed a variable (weak to strong) intensity of cytoplasmic and nuclear staining (Figure 6; 10×; clone 4C4.9, mouse monoclonal, Ventana Medical Systems, Oro Valley, AZ, USA; previously unpublished, original photo).

**Figure 7:**
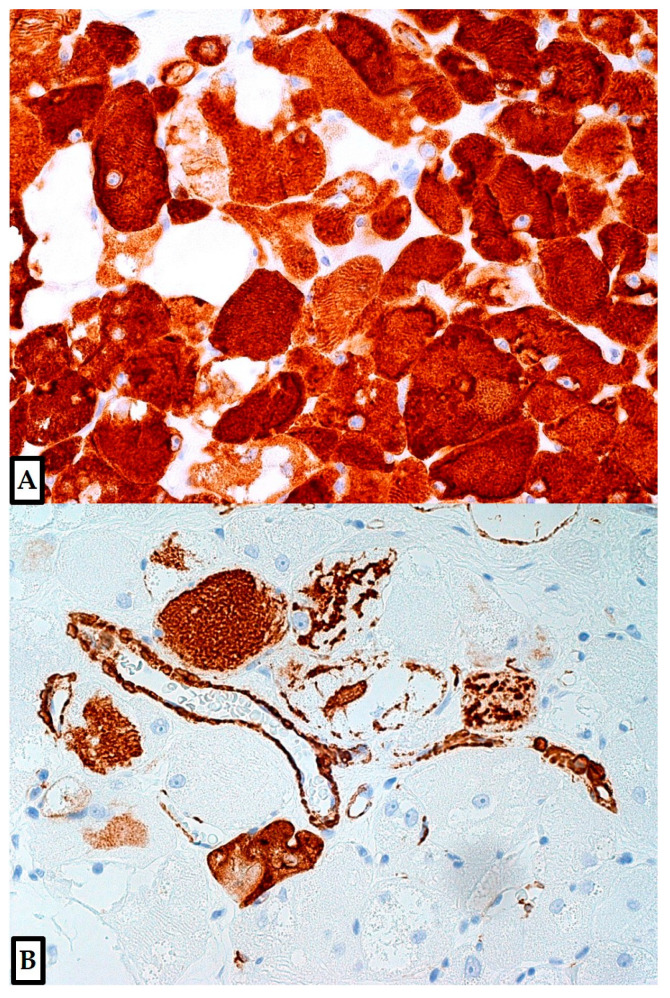
Additional markers had been previously performed, including melan-A (clone A103, mouse monoclonal, Ventana), CD68 (clones KP-1 and PG-M1, mouse monoclonal, Ventana), GFAP (clone 6F2, mouse monoclonal, Dako, Agilent Technologies, Santa Clara, CA, USA), pan-cytokeratins (clone AE1/AE3, mouse monoclonal, Dako), CEA (clone TF 3H8-1, mouse monoclonal, Ventana), calretinin (clone SP65, rabbit monoclonal, Ventana) and neurofilaments; they all resulted negative. Desmin and smooth muscle actin (SMA) were simultaneously tested in both our current and previous specimen; desmin resulted strongly positive in >98% of tumor cells ((**A**); clone DE-R-11, mouse monoclonal, Ventana Medical Systems, Oro Valley, AZ, USA; 10×; previously unpublished, original photo), while SMA was strongly expressed by 10% of RM cells (patchy positivity, mainly around capillaries/medium vessels) ((**B**); clone 1A4, mouse monoclonal, Ventana Medical Systems, Oro Valley, AZ, USA; 10×; previously unpublished, original photo). Finally, the proliferation index (Ki-67, clone 30-9, rabbit monoclonal, Ventana) was <1%. A diagnosis of an adult-type RM of the vocal cord was made. RMs are rare tumors showing skeletal muscle differentiation, distinguished as of adult (the most common), fetal, or genital type; this classification is usually done according to morphological features, despite there being a site and age predilection for each subtype [1,2,3,4,5,6,7,8,9,10,11,12,13,14,15,16,17,18,19,20,21,22,23,24,25,26,27,28,29,30,31,32]. By definition, genital RMs arise from the genitalia (vagina, vulva, cervix, paratesticular, epididymis, spermatic cord, etc.) of both sexes (8–67 years of age), entering in the differential diagnosis with various benign and malignant tumors [1,5,16,33]; on the other hand, most cases of the other two types arise in the head and neck region (pharynx, larynx, paratracheal, salivary glands, oral cavity, submandibular, soft tissues, etc.) and rarely from extremities or visceral organs (heart, bladder, stomach, etc.), with a slight male predominance [1,2,3,4,6,15,17,18,19,20,21,22,23,24,25,26,27,28,29,30,31,32]. The adult-type is the most common form (median age: 60 years; range 33–80 years), and has a male predominance [1,2,3,4,9,20,21,22,23,24,25,26,27,28,29,30,31]. Fetal RMs frequently occur in the postauricular region of newborns/children and can be associated with basal cell nevus syndrome; this syndrome is caused by *PTCH1* mutations, which can activate the hedgehog signaling [1,10,13,32,34,35]. Non-syndromic, fetal or adult RMs may also show activation of this pathway [1,34,35]. Extracardiac RMs have also been associated to Birt–Hogg–Dubé syndrome (caused by *FLCN* mutations), while cardiac rhabomyomas frequently occur in the Tuberous Sclerosis setting [1,4,6,7,8,30]. Usually described as slow-growing, painless, soft, and well-circumscribed tumors (size range: 0.5–10 cm; median size: 3 cm), adult RMs are typically benign; however, worrisome features can occur, including fast growth, multifocality (3–15%) (while fetal RMs are usually solitary) or recurrence (10–40%), even in adequately excised tumors [1,2,3,4,5,6,7,8,9,10,11,12,13,14,15,16,17,18,19,20,21,22,23,24,25,26,27,28,29,30,31,32,34,35]. While adult RMs (as in our case) typically reveal large mature rhabdomyoblasts, with scattered spider cells, fetal RMs usually show irregular bundles of immature skeletal muscle fibers, featuring fetal myotubes (round or spindle cells with occasional cross striations) arranged in a myxoid background [1,2,3,4,5,6,7,8,9,10,11,12,13,14,15,16,17,18,19,20,21,22,23,24,25,26,27,28,29,30,31,32,34,35]. Fetal RMs can be further grouped into cellular (uniform population of differentiating myoblasts) or myxoid subtypes, depending on the predominant component; mitotic activity can be relatively increased (5/50 mm^2^), especially in this subtype, in the absence of nuclear atypia, infiltrative margins, atypical mitoses or necrosis [1,10,13,32,35]. In our case, mitoses and nuclear atypia were not found. Female genital RMs are relatively small and contain loose fibrous connective tissue with spindle, polygonal, or elongated rhabdomyoblasts; male genital tumors have histological features of adult or fetal RMs [1,5,16,33]. A granular cell tumor (GCT) was erroneously diagnosed, as the tumor cells, especially at low power, show a granular eosinophilic cytoplasm due to massive accumulation of lysosomes; moreover, typical rod-like inclusions and cross striations were less evident in the previous, partially fragmented specimen. However, the cells of GCTs are usually smaller and less polygonal than those of adult-type RMs; the cell borders are usually ill-defined, sometimes indistinct, and may produce a syncytial appearance. GCT is a rare, frequently benign tumor, frequently arising in the head and neck region (about 50% of cases), especially in the tongue (25% of all cases), while the larynx is less commonly involved (6–10%) [1,36,37,38,39,40]. Other sites include deep dermis/subcutis of trunk (including breast, 5–15%) and proximal extremities, where most of the bona fide malignant cases (50% risk of metastasis) usually occur (especially in thigh) [1,36,37,38,39,40]. Visceral organs (gastro-intestinal tract, etc.) can be involved as well [1,36,37,38,39,40]. Usually solitary, they can be multifocal (10%), also involving different organ sites [1,36,37,38,39,40]. Local recurrence can be seen after incomplete excision; it could also represent an adverse prognostic factor for malignant cases, together with metastatic behavior, larger tumor size, and old age [1]. The head and neck region is less frequently involved with malignant GCTs [36,37,39,40]. GCT patients are usually adults (3rd to 6th decades); the average age of diagnosis for laryngeal GCT is 36 years [1,36,37,38,39,40]. GCTs are generally reactive for S-100, SOX10, nestin, inhibin, calretinin, CD68, CD63 (NKI/C3), and NSE; staining for melan-A is a very rare event (typically focal reactivity), while HMB-45 is usually negative [1,36,40]. Congenital granular cell lesion/epulis is a rare, benign, S-100-negative lesion that is typical of the alveolar ridge (maxilla, mandible) of newborns; the larynx is not a typical site [41,42,43]. The diffuse S-100 positivity of this case was sufficient to support the misleading hypothesis of a GCT, while muscle markers were not performed in the previous specimen. Adult RMs are usually strongly and diffusely positive for desmin, while myogenin and actin can be variably/focally expressed, as in our case [1,2,3,4,9,20,21,22,23,24,25,26,27,28,29,30,31]. Myoglobin, and MYOD1 can be positive as well [1,2,3,4,9,20,21,22,23,24,25,26,27,28,29,30,31]. GFAP and S-100 immunohistochemical expression are rarely reported in RMs, typically being focally expressed [1,3,20,21,22,23,24,25,26,27,28,29,30,31,32]. To support this consideration, we performed a brief systematic literature review, searching for (S100 OR S-100) AND (rhabdomyoma OR rhabdomyomas) in Pubmed (27 results), Scopus (46 results) and Web of Science (18 results) databases. No limitations were set. Relevant articles were obtained in full-text format and screened for additional references. The bibliographic research ended on February 12, 2022. The main limitations of our review were represented by selection biases and by the possibility that not all the articles investigating S-100 expression in RMs could have been retrieved. However, including our case, S-100 positivity was globally found in 19/34 (56%) adult-type RMs of the head and neck region [3,20,21,22,23,24,25,26,27,28,29,30,31,32]. The larger series was that of Kapadia et al. [26], who reported variable (often weak) S-100 positivity in 14/21 (67%) cases (rabbit polyclonal antibody, Dako, 1:1600 dilution). In the remaining four previously reported cases, the positivity was observed in some cells or few scattered nuclei, or it was unclear; the antibody clones were usually not reported [22,28,30,31]. Conversely, S-100 expression was confirmed in >90% of tumor cells in our case, with variable (weak to strong) intensity of staining. Finally, Kapadia et al. [32] reported that also 6/12 (50%) fetal-type RMs of the head and neck region resulted positive for S-100 (1–2+ on a scale ranging from 0 to 4, where 1+ was focal pale positivity); the antibody clone was unclear. These authors additionally specified that 5/6 (83%) cases showed S-100 expression in less differentiated (more primitive-looking), round to oval, mesenchymal cells, while there was diffuse, weak staining of the skeletal muscle fibers in the remaining case; however, it did not reach a 4+ (diffuse, intense) score [32]. Finally, especially on small biopsy material or cytologic samples taken from accessible sites, rare differential diagnoses of RMs may also include: (1) oncocytic tumors (not only oncocytomas) of the salivary glands, usually arising from the parotid, but rarely from minor salivary glands of various sites (including larynx); (2) oncocytic tumors of other origins (such as metastatic renal cell carcinoma); (3) paragangliomas (rarely involving the larynx) [44,45,46,47,48,49,50]. Oncocytomas are composed of tumor cells with abundant granular eosinophilic cytoplasm and vesicular to pyknotic nuclei, while paragangliomas show epithelioid, spindle and/or plasmocytoid cells (ovoid nuclei, fine chromatin, and inconspicuous nucleoli), arranged as single cells or in sheets [44,47,48,49]. Both tumors are typically negative for striated muscle markers, although they may occasionally show S-100 positivity (not typical) [44,47,48,49]. In conclusion, the increasing implementation of various immunohistochemical biomarkers in different tumor contexts has made wider diagnostic immunopanels available for the pathologists’ routine practice. Indeed, proper historical and novel immunostains should be tested without discounts, when indicated, to achieve the correct diagnosis, excluding other relevant differential diagnoses. Moreover, immunomarkers performed to exclude particular hypotheses may reveal unexpected results, helping to consider previously excluded or not evaluated entities. Conversely, the widespread use of inappropriate immunomarkers may result in increasing the diagnostic difficulties, in case of unpredictable results, that cannot be easily managed. The infrequent positivity of some markers in rarely tested tumors (such as S-100 in RMs) may represent misleading diagnostic pitfalls, especially when all the differential diagnoses have not been properly considered [47,48,49,50,51,52,53,54,55,56,57,58,59,60,61,62,63,64]. Morphology should be accurately evaluated, and a second opinion may be asked.
